# Linear systems analysis for laminar fMRI: Evaluating BOLD amplitude scaling for luminance contrast manipulations

**DOI:** 10.1038/s41598-020-62165-x

**Published:** 2020-03-25

**Authors:** Jelle A. van Dijk, Alessio Fracasso, Natalia Petridou, Serge O. Dumoulin

**Affiliations:** 1 Experimental Psychology, Utrecht University, Utrecht, NL Netherlands; 2 Spinoza Centre for Neuroimaging, Amsterdam, NL Netherlands; 3 Institute of Neuroscience and Psychology, University of Glasgow, Glasgow, G12 8QB UK; 4 Radiology Department, Imaging Division, Center for Image Sciences, University Medical Center Utrecht, Utrecht, NL Netherlands; 5 Experimental and Applied Psychology, VU University, Amsterdam, NL Netherlands

**Keywords:** Perception, Extrastriate cortex, Striate cortex

## Abstract

A fundamental assumption of nearly all functional magnetic resonance imaging (fMRI) analyses is that the relationship between local neuronal activity and the blood oxygenation level dependent (BOLD) signal can be described as following linear systems theory. With the advent of ultra-high field (7T and higher) MRI scanners, it has become possible to perform sub-millimeter resolution fMRI in humans. A novel and promising application of sub-millimeter fMRI is measuring responses across cortical depth, i.e. laminar imaging. However, the cortical vasculature and associated directional blood pooling towards the pial surface strongly influence the cortical depth-dependent BOLD signal, particularly for gradient-echo BOLD. This directional pooling may potentially affect BOLD linearity across cortical depth. Here we assess whether the amplitude scaling assumption for linear systems theory holds across cortical depth. For this, we use stimuli with different luminance contrasts to elicit different BOLD response amplitudes. We find that BOLD amplitude across cortical depth scales with luminance contrast, and that this scaling is identical across cortical depth. Although nonlinearities may be present for different stimulus configurations and acquisition protocols, our results suggest that the amplitude scaling assumption for linear systems theory across cortical depth holds for luminance contrast manipulations in sub-millimeter laminar BOLD fMRI.

## Introduction

The cerebral cortex consists of separate cortical regions that perform specialized computations. The first parcellation of the cortex into separate cortical regions was based on anatomical differences across cortical depth, i.e. cortical layers or laminae^1–6^, for reviews see^7,8^.

Magnetic resonance imaging (MRI) is one of the most popular techniques to study the human brain non-invasively. The recent development in static magnetic field strength to ultra-high fields of 7 Tesla and higher, has enabled researchers to investigate the human brain at a sub-millimeter (mesoscopic) scale. At this spatial resolution, it becomes possible to measure both anatomical and functional cortical depth-dependent signals that reflect contributions of different cortical layers. Sub-millimeter (laminar) functional MRI (fMRI) promises to complement anatomical measurements across cortical depth with functional properties that may indicate feed-forward and feedback processes^9,10^.

However, fMRI across cortical depth faces substantial challenges^9,11,12^. One dominant challenge relates to the cortical vascular organization. While fMRI detects hemodynamic consequences of neuronal activity^13,14^, the cortical vasculature has a specific organization across cortical layers: blood flows from pial and intracortical arteries and arterioles to the capillary bed that directly interfaces with neuronal tissue, and is then drained via venules and intracortical veins to larger pial veins at the cortical surface^15,16^. This likely explains a large part of the consistently reported finding that BOLD signal amplitude is larger at the cortical surface and systematically decreases toward deeper layers, see e.g.^9,17–31^. The vascular architecture across cortical depth has also been modeled in detail e.g.^32,33^ and poses a fundamental challenge for the analysis of laminar fMRI as blood pooling effects might differently affect the blood oxygenation level dependent (BOLD) signal in deeper layers compared to more superficial ones^34^.

**Figure 1:**
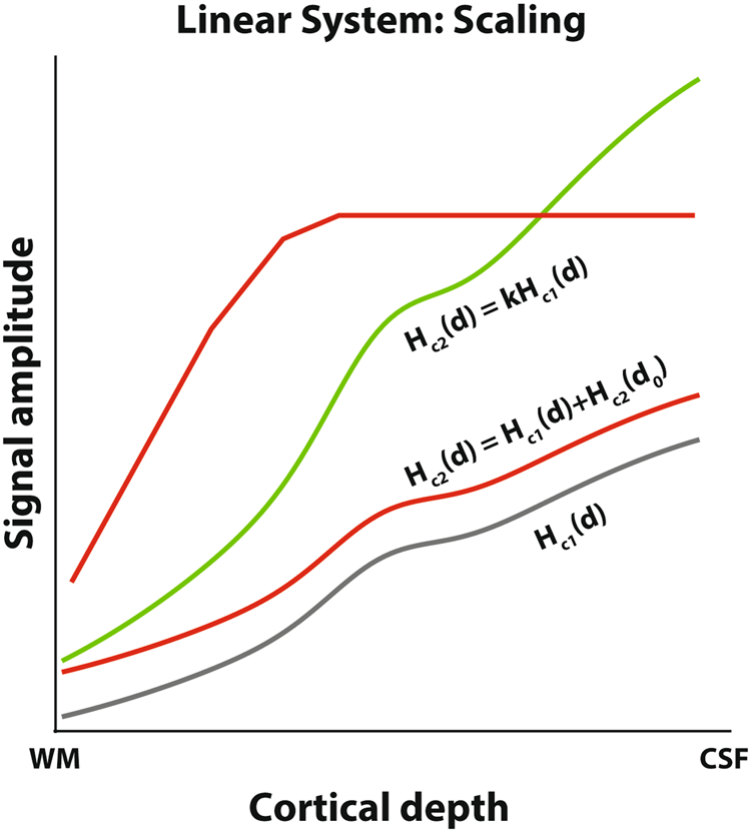
Theoretical response amplitude changes across cortical depth as luminance contrast increases. See also Eq. 4. WM = white matter, CSF = cerebrospinal fluid. Gray line: Base response amplitude across cortical depth for a given contrast, *H*_*c*_(*d*), red and green lines indicate theoretical response changes as the luminance contrast increases. The green line satisfies the scaling requirement according to linear system theory, i.e. the response increase corresponds to a multiplication of *H*_*c*_(*d*) with a scaling factor k, resulting in *kH*_*c*_(*d*). Both red lines indicate potential increases in responses that do not follow linear system theory. The lower red line corresponds to adding a response at the deepest cortical depth *d*_0_, and the resulting draining offset to the original BOLD response, *H*_*c*_(*d*) + *H*_*c2*_(*d*_0_). Although this is a linear transformation, this does not satisfy the scaling requirement for a linear system. The upper red line corresponds to a response increase that includes a maximum response. A maximum response is also a nonlinear - and biologically plausible - transformation.

Furthermore, due to the directional blood pooling across layers, hemodynamic responses from deeper cortical layers influence signals at superficial layers. For example, if there is neuronal activity in the deeper cortical depth portions only and not in the more superficial ones, a BOLD signal change would be measured at all cortical depths, due to this directional blood pooling (see Fig. 1, lowest red line). Theoretically, this could occur in absence of feedforward signals due to clinical manifestations, see e.g.^35^, or in situations where neural activity is primarily driven by feedback signals -typically arriving in the supragranular and/or infragranular depth portions, as demonstrated by Kok and colleagues^36^ and Klein *et al*.^37^.

In summary, the specific cortical vascular organization might undermine fundamental assumptions in fMRI analysis at the sub-millimeter scale. Nearly all fMRI data-analysis techniques assume that fMRI responses are linearly proportional to a local average neuronal activity over a period of time following linear systems theory^38^. For this theory to hold, two assumptions must be met: scaling and temporal additivity. These assumptions have been tested for conventional supra-millimeter fMRI for which they largely hold, provided that the stimuli used are within a defined range of stimulus parameters that is commonly used in neuroimaging experiments, see e.g.^13,38–42^; for nonlinearities, mostly found in event-related designs, see e.g.^43–49^. Based on these results at conventional resolutions, the assumptions for a linear system are expected to largely hold for responses across cortical depth. However, the laminar BOLD signal amplitudes and directional blood pooling across layers potentially violate these assumptions, as different portions across cortical depth are not independent.

Here we test whether the scaling assumption for a linear system holds in human visual cortex for BOLD amplitude across cortical depth using the most common acquisition protocol at 7T; gradient echo (GRE) BOLD fMRI. Thus, we seek to extend the BOLD linearity assumption to the cortical depth domain, specifically for amplitude scaling. If we define a system with a hemodynamic transform *L*, neuronal response over time *n*(*t*), and the hemodynamic response *H*(*t*) as output:

1 $$L[n(t)]=H(t)$$

For the scaling assumption to be satisfied, the new output must be equal to the original output, multiplied by a constant *k*:

2 $$L[kn(t)]=kL[n(t)]=kH(t)$$

On a sub-millimeter level, however, the hemodynamic response at depth *d* does not only depend on the local neuronal response, but also on responses at deeper cortical depths because of the draining effects towards the cortical surface. Thus, the hemodynamic response at depth *d* can be expressed as:

3 $${H}_{c}(d)=L{[n(t)]}_{d}+\mathop{\sum }\limits_{i={d}_{0}}^{d-1}{w}_{i}{H}_{c}(i)$$

where *H*_*c*_(*d*) is the hemodynamic response at depth *d*; $L{[n(t)]}_{d}$ is the hemodynamic transform of the local neuronal response over time at depth *d* and $\mathop{\sum }\limits_{i={d}_{0}}^{d-1}{w}_{i}{H}_{c}(i)$ reflects the draining from the sum of the hemodynamic response *H*_*c*_(*i*) at all cortical depths below depth *d*, up to the gray-white matter surface *d*_0_, each weighted by a factor *w*_*i*_^50,51^. This weighting factor represents an estimation of the draining of altered deoxyhemoglobin content and increased blood pressure from lower layers^51^. The hemodynamic response at a given depth is thus a combination of the local neuronal activity and draining from deeper layers. Therefore, the hemodynamic response is not only dependent on the neural responses at that location (or depth) but also on the neural responses at other locations (depths). This dependence might violate the assumption that the hemodynamic response is proportional to the underlying local neural activity and could drive the system nonlinear. For example, if task demands drive deeper cortical depths more actively than more superficial depths due to feedback connections^52,53^, this would result in an increase in the hemodynamic response at all depths, while there is no additional local neuronal component at most depths. This is illustrated in Fig. 1, lower red curve. This violates the scaling assumption between cortical depths.

We can express the scaling assumption across cortical depth as:

4 $${H}_{c2}(d)=k{H}_{c1}(d)$$

where *H*_*c*1_(*d*) and *H*_*c*2_(*d*) are the hemodynamic responses at each cortical depth *d*, for condition *c*1 and *c*2 respectively, and *k* is a scaling constant from one stimulus condition to another (Fig. 1, green curve), which should be constant across depth for the scaling assumption to hold across cortical depth.

We used stimuli with different luminance contrasts (i.e. sinewave gratings with 5, 20, and 80% luminance contrast) to elicit gradually increasing neuronal responses. For linear amplitude scaling across cortical depth to hold, the BOLD response amplitude across cortical depth to a higher contrast (Fig. 1, green curve) should be equal to the response profile across cortical depth for another contrast, multiplied with a scaling factor *k* that is independent of cortical depth (Fig. 1, gray curve), therefore retaining the shape but changing in amplitude. Figure 1 also shows two changes in response amplitude that do not follow linear systems theory (Fig. 1, red lines). For example, an intercept shift due to e.g. a spatially restricted increase in neuronal activation does not satisfy linear systems theory requirements (Fig. 1, lower red curve).

All subjects participated in two sessions. In one session, participants passively fixated the center of a large fixation cross, while they performed a one-back motion direction discrimination task in the other session. We find that response amplitudes increase with increasing contrast, and increase towards the cortical surface. Scaling these responses according to linear systems theory resulted in excellent overlap between responses. Our results thus support the null-hypothesis that findings from conventional (supra-millimeter) GRE BOLD fMRI extend to the cortical depth domain for stimulus luminance contrast.

## Results

### BOLD signal amplitude increases with luminance contrast and cortical depth

**Figure 2:**
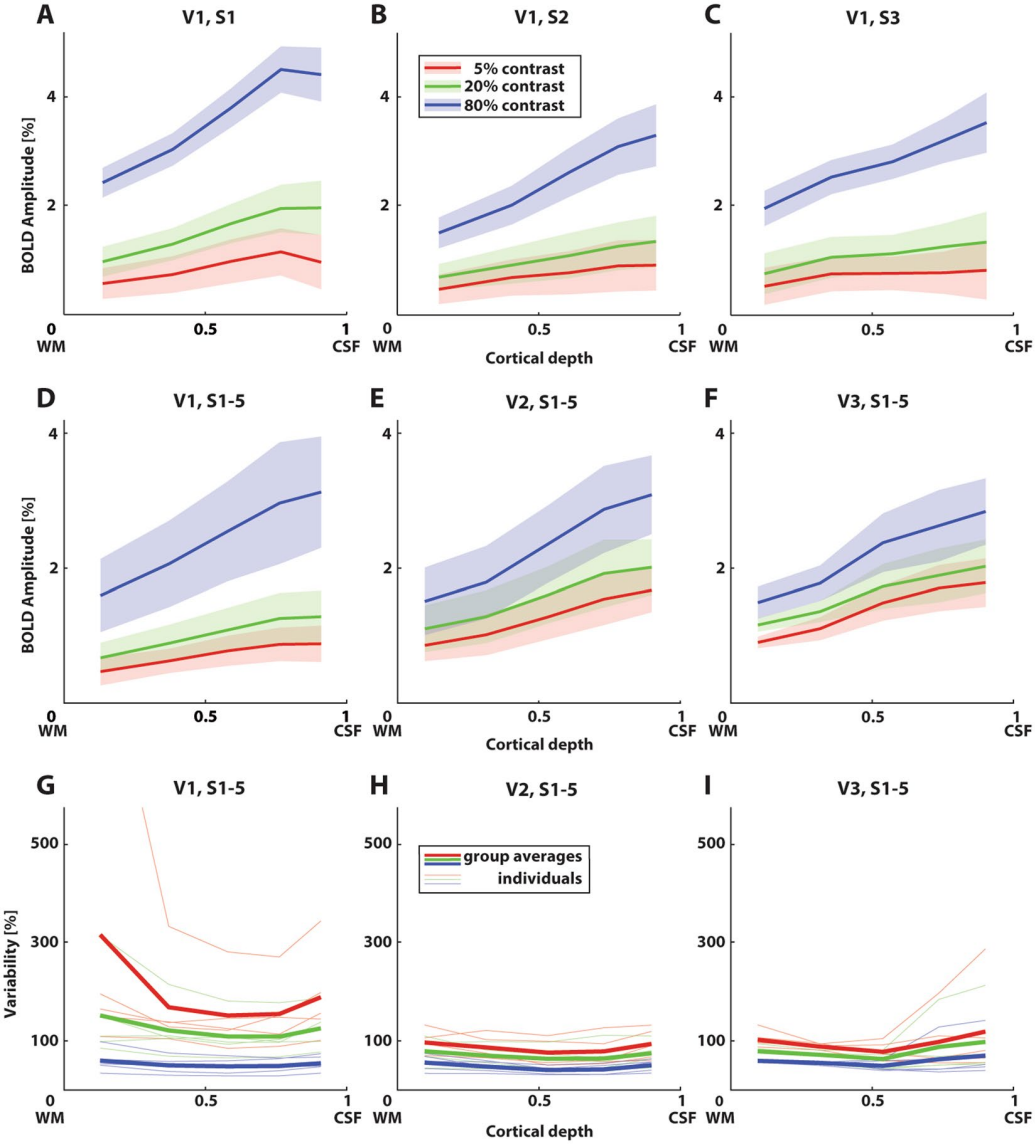
BOLD response amplitudes as a function of cortical depth and different luminance contrasts for the first session. (**A–C**) BOLD response amplitudes across cortical depth for three example participants, for visual field map V1. Error regions represent 95% confidence intervals (+/−1.96 standard errors of the mean) across repeated stimulus presentations for a region of interest, for each depth bin and luminance contrast. Panels for the other participants can be found in Fig. S2. (**D**–**F**) Group averages for visual field map V1, V2, and V3. BOLD response amplitudes increase with luminance contrast and towards the cortical surface. Error regions represent 95% confidence intervals of the mean response across depth between subjects. (**G**–**I**) Variability (standard deviation across repeated stimulus presentations divided by the mean across voxels, in percentage) over depth for visual field map V1, V2, and V3. Thin lines denote individual subject. Thick lines denote group averages. Line colors correspond to luminance contrast, as in (**A**–**F**).

For each subject, the fMRI signal increased both as a function of luminance contrast and cortical depth in V1-3 (see Fig. 2A–C for V1 for three example participants; for additional participants see Fig. S2). These signal increases are consistent with previous literature reporting increases with luminance contrast^38,39^ and cortical depth^17,21,28,54^. The latter is expected based on the cortical vasculature, as blood pooling increases towards the pial surface^15^. The 95% confidence interval across repeated stimulus presentations increased towards the cortical surface. This is expected as thermal noise scales with signal amplitude.

Next, we averaged the different subjects and observed the same signal increase (Fig. 2D–F). For example, in the central depth bin of V1 (Fig. 2D), the 5% luminance contrast elicited a response amplitude of 0.8% BOLD, whereas 20% and 80% luminance contrast elicited 1.1% BOLD and 2.6% BOLD respectively in the same depth bin. For V2 (Fig. 2E), the 5%, 20%, and 80% luminance contrast elicited a response amplitude of 1.3, 1.6, and 2.4% BOLD in the central depth bin respectively. For V3 (Fig. 2F), these values were 1.5, 1.7, and 2.4% BOLD respectively.

Variability across cortical depth (Fig. 2G–I) was expressed by dividing the standard deviation of the signal amplitude across repeated stimulus presentations at a given cortical depth bin within a given visual field map by the mean signal amplitude across repeated stimulus presentations at each depth bin within each visual field map, expressed as a percentage. This was calculated for each participant separately. Variability was highest for 5% luminance contrast responses for all visual field maps, with 80% consistently showing the lowest variability. Variability was generally lowest around the central depth bins, increasing outwards. In V1, there is a notable increase in variability near the gray-white matter surface. Additionally, variability is generally higher in V1 for 5% and 20% luminance contrast with respect to the other visual field maps. This is mainly driven by a lower BOLD response amplitude for these luminance contrasts compared to other visual field maps, as visible in Fig. 2D.

### Linear systems theory captures differences in signal amplitudes

**Figure 3:**
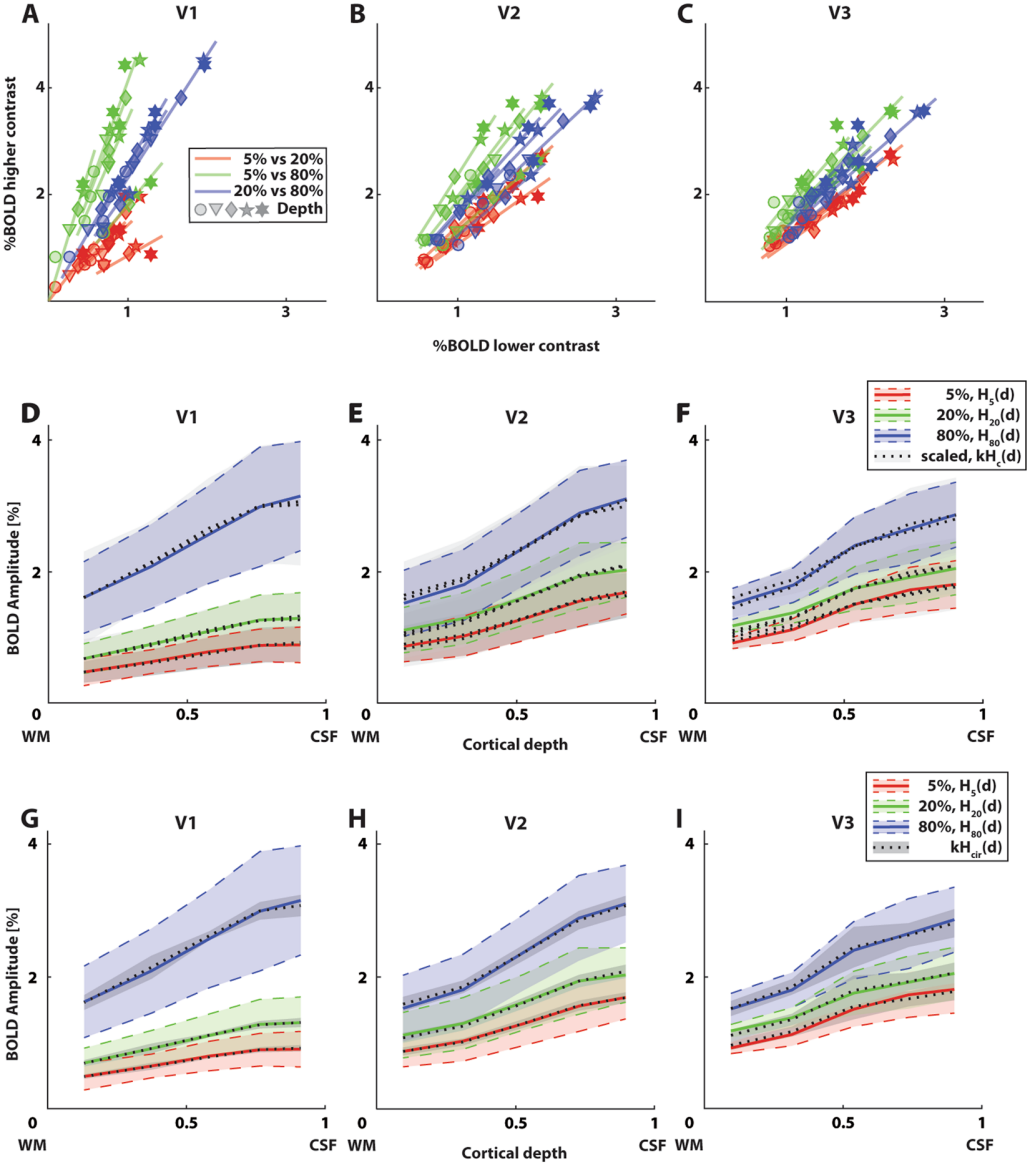
Linear systems theory captures differences in signal amplitude for the first session. (**A**–**C**) BOLD response amplitude for individual participants for V1, V2, and V3. Different marker shapes represent different depth bins. Each possible pair of two out of three luminance contrasts is plotted against each other (different colored lines), with %BOLD for the lower contrast on the x-axis, and for the higher contrast on the y-axis. Linear fits were forced to only fit a slope parameter, k from Eq. 4. These fits explained 96–99% of variance. See Fig. S2 for separate plots of each luminance contrast pair. (**D**–**F**) Measured BOLD response across cortical depth for different luminance contrasts (colored lines) and visual field maps (different panels), overlaid with scaled responses to other contrasts (dotted lines). Scaled responses represent the measured BOLD response across cortical depth for other contrasts, scaled by a scaling constant k (see Eq. 4; Table 1). These scaled responses explained 96–99% of variance. Error regions represent 95% confidence intervals (+/−1.96 standard errors of the mean) of the mean response across depth between subjects. These are scaled by a factor k for the scaled responses (see Table 1). (**G**–**I**) Measured BOLD response across cortical depth of the first session for different contrasts (colored lines) and visual field maps (different panels), overlaid with responses scaled from the contrast-independent laminar response curve (*H*_*cir*_(*d*)). These scaled responses explained 98–99% of variance. Error regions represent 95% confidence intervals of the mean contrast-independent laminar response curve. These are scaled by a factor k for the scaled responses (see Table 2).

To assess the response linearity across cortical depth, we plotted all unique combinations of BOLD responses to two different luminance contrasts across cortical depth for each individual participant (Fig. 3A–C; see Fig. S2 for separate plots for each contrast pair). If responses to a higher luminance contrast (*H*_*c*1_(*d*)) are a scaled version of a lower luminance contrast (*kH*_*c*1_(*d*), see Eq. 4), BOLD responses across cortical depth should fall on a straight line going through the origin with a slope equal to *k*. For V1, these linear fits explained an average variance of *r*^2^ = 0.99, 0.97, and 0.99 for 5% to 20%, 5% to 80%, and 20% to 80% luminance contrast scaling respectively. For V2, the average explained variance was *r*^2^ = 0.99, 0.99, and 0.98, and *r*^2^ = 0.96, 0.99, and 0.98 for V3.

To visualize the results in an alternative way, we plotted the averaged original responses (*H*_*c*1_(*d*)) and averaged scaled responses (*kH*_*c*1_(*d*), Eq. 4; Fig. 3D–F). See Table 1, left value in every cell, for all group estimates of *k*. The scaled responses were good predictors for responses at other contrasts. Both analyses indicate that the response amplitude across cortical depth elicited by one luminance contrast, was an excellent predictor for the response amplitude across cortical depth to another luminance contrast. Moreover, the scaled contrast-independent laminar response curve (*H*_*cir*_(*d*), Fig. 3G–I) explained an average variance of *r*^2^ = 0.98 for scaling to the 5% contrast response curve in V3, and explained an average variance of *r*^2^ = 0.99 for all other visual field maps and contrasts. See Table 2, left value in every cell, for the associated scaling factors *k*.

**Table 1:** Group amplitude scaling factor *k* for all unique combinations of BOLD responses to two different luminance contrasts across cortical depth.

k (session 1/session 2), individual contrast responses			
	V1	V2	V3
*H*_20_ (*d*) = *kH*_5_(*d*)	1.50/1.61	1.24/1.26	1.16/1.15
*H*_80_ (*d*) = *kH*_5_(*d*)	3.62/3.89	1.87/1.93	1.58/1.69
*H*_80_ (*d*) = *kH*_20_(*d*)	2.38/2.33	1.50/1.53	1.37/1.47

The left value in each cell represents the scaling parameter for the first session, and the right value in each cell represents the scaling parameter for the second session. Separate rows: pairs of luminance contrasts. Separate columns: visual field maps.

**Table 2:** Amplitude scaling factor k to scale the contrast-independent laminar response curve (*H*_*cir*_(*d*)) for each visual field map and session to the average responses to each luminance contrast.

*k* (session 1/session 2), contrast-independent response			
	V1	V2	V3
*H*_5_ (*d*) = *kH*_cir_(*d*)	0.90/0.88	1.67/1.56	1.77/1.81
*H*_20_ (*d*) = *kH*_cir_(*d*)	1.28/1.32	2.07/1.97	2.04/2.08
*H*_80_ (*d*) = *kH*_cir_(*d*)	3.06/3.05	3.07/3.01	2.80/3.04

The left value in each cell represents the scaling parameter for the first session, and the right value in each cell represents the scaling parameter for the second session. Separate rows: pairs of luminance contrasts. Separate columns: visual field maps.

### Linear amplitude scaling generalizes across sessions

Next, we assessed whether these results generalized between sessions. In the second session, participants had to detect when the motion direction of the stimulus was identical on two consecutive presentations. The average discriminability (d’) was 2.2, indicating a detection performance of well above chance level (d’ of S1-5 were 2.6, 2.2, 2.1, 2.3, and 1.8 respectively, minimum d’ for any individual run is 1.4).

**Figure 4:**
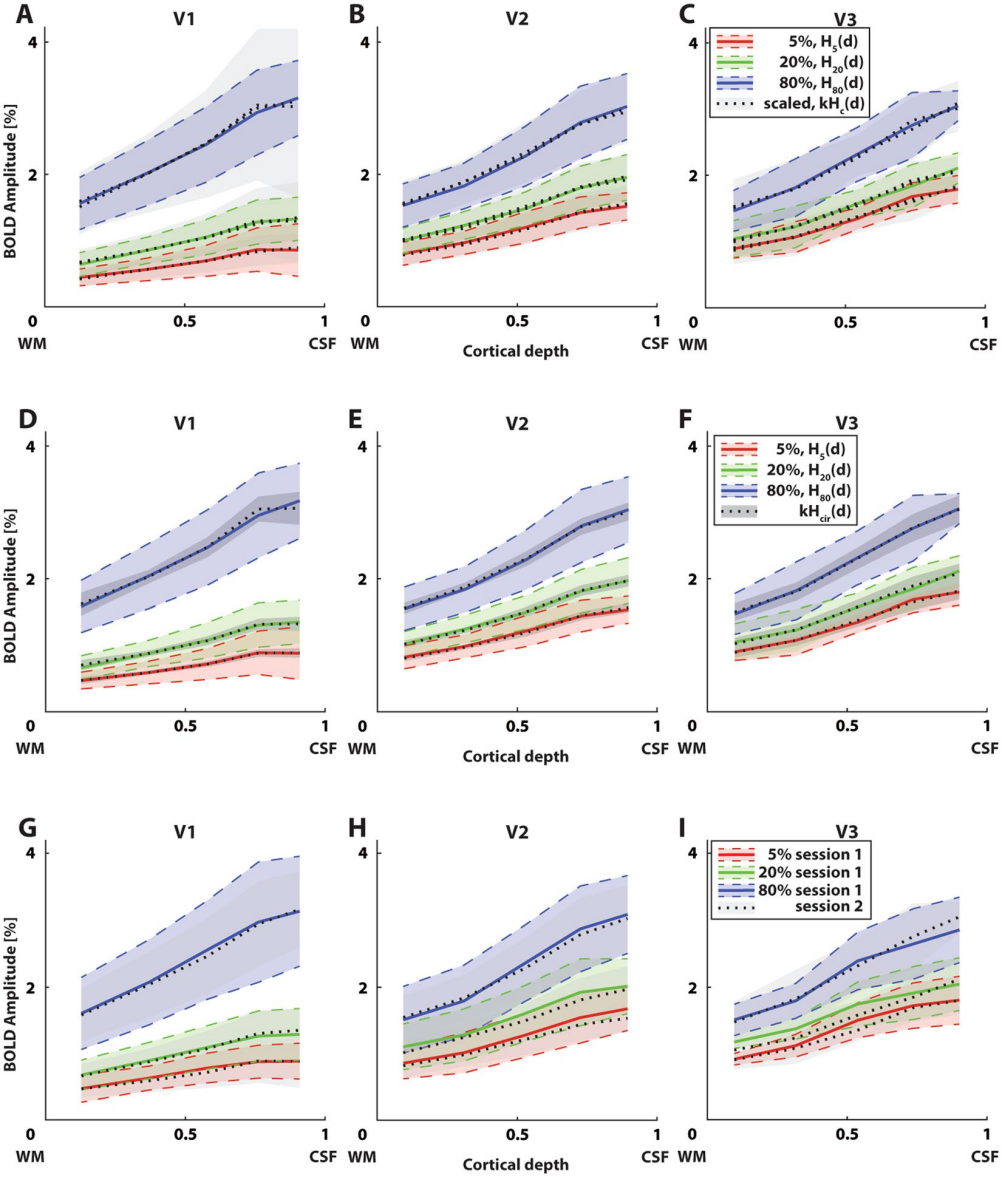
BOLD response amplitude and amplitude scaling for the second session are very similar to the first session. (**A**–**C**) Measured BOLD response across cortical depth of the second session for different contrasts (colored lines) and visual field maps (different panels), overlaid with scaled responses to other contrasts (dashed lines). These scaled responses explained 96–99% of variance. Error regions represent 95% confidence intervals of the mean response across depth between subjects. These are scaled by a factor k for the scaled responses (see Table 1). (**D**–**F**) Measured BOLD response across cortical depth of the second session for different contrasts (colored lines) and visual field maps (different panels), overlaid with responses scaled from the contrast-independent laminar response curve (*H*_*cir*_(*d*)). These scaled responses explained 99% of variance. Error regions represent 95% confidence intervals of the mean contrast-independent laminar response curve. These are scaled by a factor k for the scaled responses (see Table 2). (**G**–**I**) Group average BOLD response amplitudes across cortical depth for the first session (solid lines) and second session (dotted lines, grayscale). Error regions represent 95% confidence intervals of the mean response across depth between subjects. Responses for both sessions matched almost perfectly (explained variance 99%).

Similar to Fig. 3D–F, we plotted the averaged original responses (*H*_*c*1_(*d*)) and averaged scaled responses (*kH*_*c*1_(*d*), Eq. 4; Fig. 4A–C). In the central depth bin of V1 (Fig. 4A), the 5% luminance contrast elicited a response amplitude of 0.7% BOLD, whereas 20% and 80% luminance contrast elicited 1.1% BOLD and 2.5% BOLD respectively in the same depth bin. For V2 (Fig. 4B), the 5%, 20%, and 80% luminance contrast elicited a response amplitude of 1.2, 1.5, and 2.3% BOLD in the central depth bin respectively. For V3 (Fig. 4C), these values were 1.3, 1.6, and 2.3% BOLD respectively. See Table 1, right value in every cell, for all group estimates of *k*. The scaled responses were excellent predictors for responses at other contrasts. For V1, these scaled responses explained an average variance of *r*^2^ = 0.98, 0.96, and 0.99 for 5% to 20%, 5% to 80%, and 20% to 80% luminance contrast scaling respectively. For V2, the average explained variance was *r*^2^ = 0.99, 0.99, and 0.99, and *r*^2^ = 0.98, 0.99, and 0.99 for V3. The scaled contrast-independent laminar response curve (*H*_*cir*_(*d*), Fig. 4D–F) explained an average variance of *r*^2^ = 0.99 for all visual field maps and contrasts. See Table 2, right value in every cell, for the associated scaling factors *k*.

Furthermore, we overlaid the responses across cortical depth from each session (Fig. 4G–I). These responses were near identical (*r*^2^ = 0.99 for all contrasts and visual field maps), indicating that the reliability between sessions of luminance contrast responses across cortical depth is excellent.

## Discussion

We assessed whether the scaling assumption for a linear system holds for BOLD amplitude across cortical depth. We evaluated BOLD fMRI responses across cortical depth elicited by viewing moving sinewave gratings with 5%, 20%, and 80% luminance contrast. We find that BOLD responses vary as a function of both luminance contrast and cortical depth. Additionally, we find that the variability of the BOLD response amplitude decreases with increasing luminance contrast, and that variability is highest in V1. Moreover, we find that scaled BOLD responses across cortical depth - elicited by viewing one luminance contrast- are excellent predictors (average explained variance *r*^2^ = 0.96–0.99) for responses across cortical depth to another luminance contrast, supporting the validity of the amplitude scaling assumption for BOLD amplitude across cortical depth. We do not find differences between scanning sessions, indicating that the robustness of the amplitude scaling assumption for the tested visual stimulus across cortical depth is stable over time.

Variability across cortical depth decreased with increasing luminance contrast, with 5% luminance contrast showing the highest variability across cortical depth for all visual field maps. This can be attributed to consistently lower signal amplitudes than for other luminance contrasts, with comparable variance (see Fig. 2A–C, Fig. S2). Generally, variability showed a U-shaped profile across cortical depth, being lowest around the central depth bins and increasing towards the WM/CSF and WM/GM borders (see Fig. 2G–I). However, as physiological and thermal noise may have different layer dependence, variability profiles across depth and the specific values measured here are likely to vary with parameters such as functional resolution, distance of the measured tissue to the radio frequency coils, and task-related signal amplitude.

Several recent studies have investigated the laminar response profiles focusing on the underlying neurovascular coupling in humans^17,19–22,31,55–62^; non-human primates^30,63–67^; and other mammals^29,68–74^. All these studies have focused on the feasibility of laminar imaging. In addition, a few studies have employed sub-millimeter fMRI for systems and cognitive neuroscience questions^36,51,54,60,75–82^. However, these latter studies have largely avoided the interpretation of BOLD amplitude measures, as this type of measure is susceptible to blood pooling effects across cortical depth. Here we provide data for the usability of BOLD fMRI amplitude measurements across cortical depth and the amplitude scaling linearity assumption that underlies many fMRI analyses.

Here we used a T2*-weighted, gradient-echo (GRE) based 3D-EPI acquisition to collect the functional data. This type of acquisition is sensitive to the microvasculature, but also to larger vessels (macro-vasculature; e.g. draining veins) and is thus affected by blood-pooling effects across cortical depth. Therefore, a signal elicited at a deeper cortical depth affects the measured signal at more superficial cortical depths, which results in less laminar specificity and the commonly observed signal increase in that direction, also found in this study. These effects could be corrected for in the analysis, for instance by implementing spatial correction approaches as proposed by Markuerkiaga and colleagues^83^ and Marquardt and colleagues^51^, or potentially by implementing more elaborate models of BOLD responses at different cortical depths that better capture general spatiotemporal properties of the hemodynamic signal, and the dynamics of capillary, arterial, and venous effects^23,32,34,84–87^ with respect to more traditional hemodynamic models^88,89^. We observed a relative decrease in BOLD amplitude for the most superficial depth bin in several visual field maps and for several luminance contrasts. These could be due to partial volume artifacts, and because the microvasculature is less dense near the cortical surface^15^.

One could argue that the increase in BOLD amplitude towards the cortical surface overshadows any cortical depth-dependent specificity in sub-millimeter GRE-based fMRI, i.e. the depth-dependent signal may arise from partial volume effects of the larger vessels at superficial depths only and signals may decrease as a function of the distance from these depths. The BOLD signal stemming from the pial veins may contribute to the trend found here. However, biophysical models outline that in the worst-case scenario, extravascular contamination from the veins at the pial surface falls off four-fold at a distance of half the vessel diameter, thus rapidly decreasing when moving away from the cortical surface^90^. Additionally, it has been shown that signals from central layers can be separated from those of pial veins, presumably depending on the venous topology at the pial surface^91^.

It is possible that the different vascular compartments across cortical depth have different (non)linear contributions to the measured BOLD signal at different luminance contrasts. Contributions from either the macro-vasculature (draining veins) or the micro-vasculature (capillaries and small venules) may be nonlinear while contributions from the other vascular compartment is linear, or both contributions may be nonlinear but cancel each other out. Previous findings have suggested that non-linear contributions from the micro-vasculature are less likely^92^. Thus, the most plausible case is that macro-vascular contributions are nonlinear and micro-vascular contributions are linear. We speculate that given the sensitivity of GRE-BOLD fMRI to the former, one would expect that nonlinearities across cortical depth resulting from the macro-vasculature would have been detected here. We speculate that an increase in spatial resolution would not yield considerably different results. One could also argue that the micro-vasculature can exhibit nonlinearities, while the macro-vasculature exhibits linear contributions to BOLD signal amplitude across cortical depth, although this scenario is less likely. However, if this were the case, GRE-BOLD fMRI would be able to pick up on nonlinearities in the micro-vasculature, especially closer to the gray-white matter surface as the capillary density is high at that compartment^15^. These would be detectable as nonlinearities at deeper and intermediate cortical depths, as compared to superficial depths, as these are most contaminated by macro-vascular contributions. If both the macro-vascular and micro-vascular contributions are nonlinear but cancel each other out, it would be hard to separate these contributions using GRE-BOLD fMRI. However, this would mean that linearity can still be assumed for most practical applications. Interestingly, the model for laminar BOLD responses by Havlicek and Uludag^87^ predicts linear scaling of BOLD amplitude across cortical depth, although the model is nonlinear. This may thus tie in with the above possibility of combined nonlinear responses resulting in linear measurements. All in all, linearity is not a feature that is only expected of GRE-BOLD fMRI, but also for other acquisition sequences at sub-millimeter resolutions, e.g. spin echo-based acquisitions, 3D-GRASE^93^, and VASO^60,69,94^. If anything, it is expected that GRE-BOLD fMRI is more sensitive to nonlinear macro-vascular contributions than most (if not all) other sub-millimeter acquisition protocols.

Although the explained variance for the amplitude scaling is extremely high, there are minor deviations from linearity. These deviations are also apparent in Figs. 3A–F, and 4D–F. The deviations from linearity are more apparent at the highest luminance contrast, hinting at a potential increase in nonlinearity near the higher end of stimulus parameter space.

For a more complete evaluation of linear systems theory for BOLD amplitude across cortical depth in the visual domain, stimuli varying in high and other low-level domains such as spatial frequency, as well as the temporal additivity assumption for a linear system should also be investigated. However, the current stimuli span a wide range of stimulus energy, also using luminance contrasts at the edge of parameter space (5% and 80%). Thus, if the BOLD amplitude across cortical depth were nonlinearly scaled between different stimulus energy, we would have been able to capture this. Moreover, contrast manipulations are frequently used in visual neuroscience, and were originally used for the assessment of the scaling assumption at supra-millimeter resolutions^38,39,95^.

Havlicek and Uludag suggest that the scaling assumption may be violated in certain cases, for example when there is a significant difference in neuronal activation or cerebral blood flow between cortical layers^87^ (also see Eq. 3). Arguably, this violation of linearity would be most visible using a stimulus paradigm that activates only a subset of layers, yet can be varied systematically, and ideally elicits responses across cortical depth that have some distinct feature, rather than the monotonic increases as found here. Potential candidates for this stimulus paradigm might be the Kanizsa triangle visual illusion or spatial attention manipulation paradigms, as these stimuli elicit a distinct response in deep cortical layers due to top-down feedback^36,37^. A viable manipulation may be illusion strength or task difficulty of a spatial attention task, though it is unknown if and how top-down feedback scales. Here, we show that linear scaling holds for luminance contrast manipulations. However, this might not hold for other stimulus paradigms, especially ones involving stimuli that show a more distinct profile across layers.

The current results can be generalized for the visual domain along different contrast levels, while the scaling assumption remains to be validated for other modalities and measurements of contrast energy. Our data do not allow for the investigation of different contributions to the generation of the BOLD signal, so neural to vascular/cortical blood flow (CBF) and vascular to BOLD contributions cannot be disentangled with the current experimental design. Additionally, because of the currently used pooling of large numbers of voxels using a region of interest-based approach, we cannot exclude that local pockets of non-linearity might be observed within a region of interest (here: visual field map). Furthermore, while the current results evaluate BOLD amplitude scaling during ‘steady-state’, scaling may be different or even violated when looking at specific phases of the BOLD response (e.g. early, late, and post-stimulus undershoot). This specific assessment is beyond the scope of the current experiment, as acquisition and stimulus presentation parameters are suboptimal for this. In summary, our results do provide evidence for the linearity of steady-state BOLD amplitude across cortical depth as a function of luminance contrast for region of interest-based approaches. The temporal additivity assumption for a linear system was not currently assessed as the presently used volume acquisition time (4 s) results in too sparse a sampling of the fMRI time-series to assess this property. At the current resolution (0.7 mm isotropic voxels), this is the fastest our system could collect data. Thus, only one out of two assumptions for a linear system across cortical depth has been evaluated thus far.

To our knowledge, response amplitude to different luminance contrasts is only assessed by one other laminar fMRI publication to date. In that study, Marquardt and colleagues^51^ assess the cortical depth profiles of luminance contrast responses, but do not assess luminance contrast linearity of BOLD amplitude across cortical depth. Instead, they propose a novel spatial deconvolution approach to correct for the directional component of the intracortical vasculature^51^. The range of their measured signal amplitudes is similar to the current findings. They also use a GRE-based EPI sequence with the same resolution, albeit with a shorter volume acquisition time (2.94 s versus 4 s here) and more coverage (52 slices versus 34 slices in the present study). Upon simple visual inspection, both their deconvolved and original results are likely to fit with the current findings, providing further evidence that the scaling assumption for a linear system holds across cortical depth for luminance contrast manipulations.

We do not find any evidence for significant changes in signal amplitude across cortical depth between sessions, even though the second session included a stimulus-related task. This appears to be in contradiction with previous studies that have shown attention-related differences for supra-millimeter fMRI (see e.g.^96,97^); across cortical depth in e.g. population receptive field center positions^37^; or suggest such differences due to e.g. a lack of feed-forward input in patients with macular degeneration^35^. Potential explanations for the apparent lack of task-driven changes in our study are two-fold. First, evidence suggests that attentional signal modulation in early visual cortex is reduced with respect to extrastriate visual field maps^37,96,98–100^. Moreover, Klein *et al*.^37^ focused on measures that do not depend on signal amplitudes. When comparing signal amplitudes, they found little modulation of BOLD amplitude between different attention conditions. Additionally, the one-back task used in the present study required very little attentional resources to carry out correctly as the events were sparse, and the stimulus was very salient. We therefore argue that attentional modulation did not play a role in the present study.

## Conclusion

We provide evidence that the scaling assumption for linear systems theory can be met for BOLD amplitude measures across cortical depth, i.e. responses scale with luminance contrast, and this amplitude scaling is identical across cortical depth. Although nonlinearities may be present for different stimulus configurations and acquisition protocols, the BOLD linearity assumption for amplitude scaling commonly employed for conventional fMRI analysis extends to the cortical depth domain for luminance contrast manipulations.

## Methods

### Participants

Five participants (all male, age range 23–44 years) took part in the experiment. One participant was left-handed. All participants had normal or corrected-to-normal visual acuity and were experienced with the MRI environment. Informed consent was acquired from all participants. All experimental procedures were conducted in accordance to the Declaration of Helsinki and approved by the ethics committee of the University Medical Center Utrecht.

### Stimuli

Stimuli were presented on a 32′′ LCD screen, specifically designed for use in an MRI environment^101^. The screen resolution was 1920 × 1080 pixels, with an active area of 69.8 × 39.3 cm and a refresh rate of 120 Hz, with a built-in linear luminance look-up table. The display was positioned at the end of the bore, and viewed via a mirror, at a total viewing distance of 220 cm. The stimulus diameter subtended 10.2 degrees of visual angle (deg).

**Figure 5:**
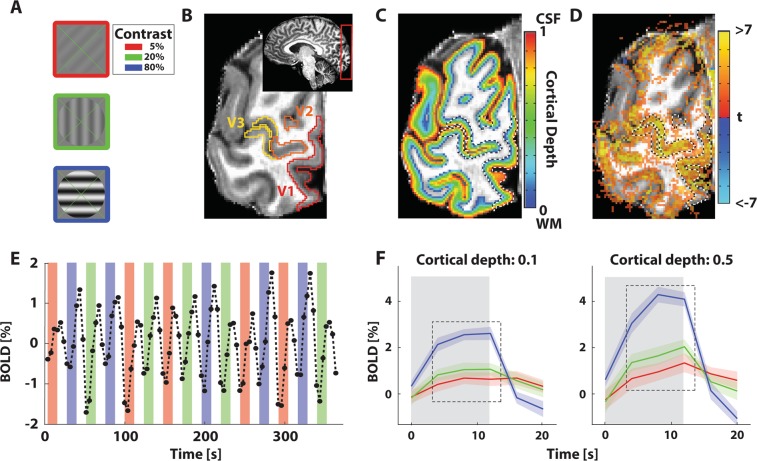
Stimulus design and processing steps. (**A**) Example stimuli with 5% (red border), 20% (green border), and 80% (blue border) luminance contrast. (**B**) Example anatomical slice with visual field map definition overlay. Inset: slab positioning for functional data acquisition. (**C**) Anatomical slice with volume-preserving depth map (colored), and visual field map (dashed lines) overlay. (**D**) Anatomical slice with t-statistical map (colored), and visual field map (dashed lines) overlay. This illustrates the accuracy of the registration. This statistical map is only used for illustrative purposes. (**E**) Example time-series. Colors correspond to luminance contrast presentations with the same color-code as (**A**). Colored bars represent when the stimulus was shown, transparent bars represent the presentation of mean-luminance baseline. (**F**) Averaged block responses at relative cortical depth 0.1 and 0.5, where 0 is the gray/white matter border, and 1 is the gray matter/cerebrospinal fluid border. The line color corresponds to responses elicited by the luminance contrasts as in (**A**). Error regions represent 95% confidence intervals (+/−1.96 standard errors) of the mean across repeated stimulus presentations for V1. Gray rectangle represents when a stimulus was presented. Dashed box represents the time points included in the analysis.

All stimuli were generated with MATLAB (Mathworks. Matlab; 2015b) using the Psychophysics Toolbox^102,103^. A green fixation cross (0.1 deg in diameter) stretching from corner to corner throughout the center 10.2 deg, was presented at all times. The stimuli consisted of sinewave gratings (0.5 cycles per degree) oriented in any of 4 possible directions (0–135 deg in 45 deg steps; Fig. 5A; similar to^104^). The gratings were moving perpendicular to the orientation of the bars at 1.76 deg per second and limited by a circular aperture with 5.1 deg radius. Movement was either towards the left, or towards the right edge of the screen. The luminance contrast of the gratings was 5, 20, or 80%. These contrasts largely lie within the approximately linear range of stimulus space^95^.

Stimuli and fixation cross were presented for 12 seconds, followed by a 12-second period of mean luminance baseline with the fixation cross. During the 12 seconds of stimulus presentation, stimuli were shown each time for 750 ms, followed by 250 ms of mean luminance. The subsequent presentation randomly moved in the same motion direction as the previous presentation (12.5% chance), or not. No more than two consecutive presentations could have the same motion direction. Each block contained a stimulus of only 1 luminance contrast. Each run consisted of 15 blocks (5 blocks of each contrast, presented in a semi-randomized order). As the repetition time of the functional volumes was very long and averaging between session is undesirable at sub-millimeter resolution, this design was the most reasonable.

### Task

Participants completed two sessions. Each session consisted of 6 or 7 runs. The same stimuli were used during both sessions. During one session, participants were instructed only to fixate the center of the fixation cross. To assess the stability of amplitude scaling between sessions, subjects performed a one-back motion direction discrimination task on the other session. During this session, participants were instructed to fixate the center of the fixation cross and indicate whenever two consecutive stimulus presentations had the same motion direction by means of a button press.

### Visual field maps definition

Visual field maps were acquired in separate scanning sessions, using near-identical procedures as in previous studies, e.g.^105^. All visual field maps were restricted to 5.1 degrees eccentricity -the radius of the stimulus in this study- based on eccentricity estimates following the population receptive field (pRF) approach^105^. All data were collected using a Philips Achieva 7T scanner (Philips, Best, the Netherlands) using a dual-channel volume transmit coil and a 32-channel receive coil (Nova Medical, MA, USA), using a 2D-EPI sequence. For participants S1, S4, and S5, visual field mapping data were collected using a traversing bar-stimulus as in^105^, but with gray-scale natural images as carrier. Functional data were acquired at a 1.8 mm isotropic resolution, with a TR/TE = 1500/25 ms, and 248 time points per run. Three runs were collected for each participant. For S2, and S3, identical stimuli were used as by Dumoulin & Wandell. For S2, 8 functional runs were collected at a resolution of 2 mm isotropic, and TR/TE = 1500/25 ms (248 time points per run). For S3, 7 functional runs were collected at a resolution of 1.8 mm isotropic, TR/TE = 2100/25 ms (182 time points per run). Visual field maps for visual areas V1, V2, and V3 were defined for all participants (Fig. 5B).

### High resolution MRI and fMRI acquisition

High resolution anatomical and functional data were acquired using a Philips Achieva 7T scanner with a maximum gradient strength of 40mT/m and a slew rate of 200T/m/s (Philips, Best, The Netherlands). A dual-channel volume transmit coil was used for all scans (Nova Medical, MA, USA). A 32-channel receive coil (Nova Medical, MA, USA) was used for all anatomical scans, while two custom-built 16-channel high-density surface receive arrays were used for all functional scans^106^ (MRCoils BV). These surface arrays were positioned adjacent such that the two arrays touched each other lengthwise but did not overlap. Participants were positioned such that their external occipital protuberance was approximately aligned with the center between the arrays, at the height of the most distal receive elements from the isocenter.

Anatomical data were acquired using an MP2RAGE sequence^107^. Whole-brain sequence parameters were: TI1 = 800 ms, TI2 = 2700 ms, TR_MP2RAGE_ = 5500 ms, TR/TE = 6.2/2.3 ms, flip angle α1 = 7, and α2 = 5, bandwidth = 403.7 Hz/pixel, acceleration factor using SENSE encoding = 3.5 ×1.3 (RL and AP respectively), resolution = 0.64 mm isotropic, total scan time 9 min 57 s.

Functional data were acquired with a T2*-weighted 3-dimensional multi-shot EPI (3D- EPI, two shots per slice, 34 slices, 68 shots overall). The sequence parameters were: TR/TE = 59/28 ms, flip angle: 20°, acceleration factor using SENSE encoding: 3.5 (right-left) × 1.3 (anterior-posterior), echo planar factor: 27, bandwidth (phase-encode): 19.1 Hz/pixel, voxel size = 0.70 mm isotropic, FOV = 131 (right-left) × 120 (feet-head) × 24 (anterior-posterior) mm, 34 coronal slices, and 28% oversampling in the slice direction. Functional volumes were acquired every 4 s and functional scans were each 91 time frames (364 s) in duration.

Additionally, we acquired ‘topup’ functional data with reversed right-left phase-encoding and geometrical distortions in opposite directions from the original functional data^108^. Images with opposite phase encoding polarities were extracted, and susceptibility induced distortion was estimated using nonlinear warping to estimate the undistorted mid-point between the two volumes in AFNI^109^. Five volumes were acquired per topup run. For each session, 6 or 7 functional and accompanying topup runs were acquired for each participant.

### Pre-processing: functional data

The fMRI data pre-processing was performed using AFNI. First, motion parameters between runs were computed by aligning the first volumes for each run to the first volume of the first run. Subsequently, motion parameters within runs were calculated by aligning each volume within a run to the first volume of that run. The individual motion-corrected runs were then despiked, scaled, and detrended. During the scaling step, the time-series for each run and every voxel were converted to percentage BOLD by dividing the signal of each voxel by its temporal mean, multiplying the resulting signal with 100, and subsequently subtracting 100 to ensure that the mean temporal response for each voxel was zero percent. The detrending step was preformed using the AFNI function 3dDtrend with a fourth-degree polynomial detrending. A warp field to correct for geometric distortions in the 3D-EPIs was calculated using a nonlinear transformation, performed on the original (non-motion corrected, scaled, despiked, or detrended) volumes. For this, the first 5 volumes of the 3D-EPI for each run, and the 5 volumes of each topup 3D-EPI run were used. As the 3D-EPI volumes have the opposite distortions in the phase-encoding direction from the topup 3D-EPI, the undistorted 3D-EPI volume is the halfway warping between the two extremes^108^.

Next, the motion estimates and warp field results were combined to calculate the 3D-EPI mean image, averaging over all warped and motion corrected volumes between runs and collapsing over all time points. This mean EPI image was then registered to the anatomical data. This process involved several steps. First, the anatomy was clipped so that only the occipital lobe was left. Then the anatomy and mean EPI image were brought into the same space by aligning their respective centers of mass. Next, the ‘Nudge dataset’ plugin in AFNI was used to provide a good starting point for the automated registration. This registration consisted of two affine transformations, using the local Pearson correlation as cost function^110^. The first one allowed for a maximum shift and rotation of 3 mm. The second iteration allowed for a maximum shift and rotation of 1 mm. The transformation matrices for the manual and automated steps were joint into a single affine matrix. To reduce the number of interpolation steps we applied this registration matrix along with the warp field (see previous paragraph) in a single step to each motion-corrected, despiked, scaled, and detrended run (see previous paragraph) to align these volumes to the registered mean EPI image. For this, we used the AFNI function 3dNwarpApply with nearest neighbor interpolation. Then, the resulting volumes were resampled into anatomy space to allow for accurate cortical depth calculation, using nearest neighbor interpolation. This procedure resulted in registered time-series for each run. All in all, our procedure involved a total of two spatial interpolation steps to get from the motion-corrected, despiked, scaled, and detrended runs to the registered, topup-corrected time-series for each run in anatomy space.

### Pre-processing: anatomical data

The MP2RAGE anatomical images were segmented using the CBS-tools plugin^111^ (www.nitrc.org/projects/cbs-tools/) in MIPAV (http://mipav.cit.nih.gov/), and subsequently manually optimized in 3D Slicer^112^. Next, a volume-preserving distance map between the gray matter-white matter (GM/WM) border and the gray matter-cerebrospinal fluid (GM/CSF) border was computed^113^ in 6 separate level-set volumes. This approach takes into account cortical folding in the depth estimation and is implemented in CBS-tools. These level sets were projected on the 3D-EPI space for subsequent laminar analysis (Fig. 5C).

### Analysis

#### BOLD across cortical depth

For each participant, visual field map (V1, V2 and V3) and luminance contrast, we took each registered, topup-corrected time-series (Fig. 5E) and divided these into 24 s blocks, each starting at stimulus onset. The volume-preserving distance map was divided in 5 quantiles, spanning from gray matter/white matter (GM/WM) border to gray matter/cerebrospinal fluid (GM/CSF) border. We calculated the mean percentage BOLD signal change for the response amplitude of the second, third, and fourth sampling point in a block (at 4, 8, and 12 s after stimulus onset respectively; see Fig. 5F, dashed box), and the 95% confidence interval (+/− 1.96 standard errors of the mean) across repeated stimulus presentations for each depth bin and luminance contrast. Note that this approach ignores the depth-dependent differences in temporal BOLD response characteristics, see e.g.^21,114^. As the temporal resolution in this experiment is 4 s, we only sample a few points of the response curve, and these temporal BOLD response differences are too small to effectively pick up at this temporal resolution. Moreover, averaging over the sampling points further reduces the presence of small temporal differences. Initial analysis using a basic general linear model (GLM) with luminance contrast as independent variable, yielded near identical results. This type of analysis also takes the post-stimulus undershoot into account. However, as the basic assumptions for a GLM include that the system that is being tested adheres to linear systems theory, this analysis is inherently circular and therefore not used in this study. We calculated the variability across repeated stimulus presentations of the BOLD response amplitudes to each luminance contrast for each visual field map by dividing the across repeated stimulus presentations standard deviation by the mean signal amplitude across repeated stimulus presentations at each depth bin. These estimates were then averaged over participants (Fig. 2G–I). Similar measures were previously used for depth-dependent fMRI by Kim & Ress^28^.

It is commonly observed that the relationship between luminance contrast and BOLD amplitude is nonlinear^13,95^. While not the main focus of the current study, we have plotted this relationship for three cortical depth bins for all regions of interest and sessions, generally replicating this finding (Fig. S3). We are thus dealing with a nonlinear transformation between stimulus intensity and CBF, and other nonlinear transformations between CBF and BOLD^115^.

#### Linearity assessment

To assess the response linearity across cortical depth, we first plotted all unique combinations of BOLD amplitudes across cortical depth (*H*_*c*1_(*d*) and *H*_*c*2_(*d*)) to two out of three different contrasts across cortical depth against each other for each individual participant and visual field map (5% vs 20%, 20% vs 80%, and 5% vs 80% grating contrast for V1, V2, and V3; see Fig. 3A–C for group averages, and Fig. S2 for individual contrast pairs). We then used a linear fit to estimate the best fitting line to the BOLD amplitudes from each of the combinations of BOLD responses across cortical depth mentioned above. Only a slope parameter was estimated with this linear fit, as is required to satisfy the linear amplitude scaling assumption. This was done for all individual participants, and repeated for V1, V2, and V3. This estimated slope of the best fitting line then corresponds to the scaling constant *k* in Eq. 4.

Next, we overlaid the responses to different contrasts for each participant and region of interest by scaling each stimulus contrast response (*H*_*c*2_(*d*)) by a constant *k* to fit other measured luminance contrast responses across depth (*H*_*c*1_(*d*) see Figs. 3D–F and 4A–C for group averages). As there were multiple luminance contrasts, multiple scaling constants were estimated, one for each combination of *H*_*c*1_(*d*) and *H*_*c*2_(*d*). These scaling constants were estimated using a regression for each luminance contrast pair, fitting only a slope parameter to scale one luminance contrast response amplitude across depth to the other, thus omitting the intercept estimation. Then, we calculated the explained variance of the original responses (*H*_*c*1_(*d*)) based on the scaled responses to the other contrasts *kH*_*c*2_(*d*) to assess the degree of linearity of the responses across cortical depth. Explained variance was calculated for each pair of contrasts (one original, and one scaled) separately. Note that the calculated amplitude scaling constants and explained variance using this approach, and the approach used in the previous paragraph, are identical. However, the results from the analysis in this paragraph are displayed at the group level. As such, the two approaches are complementary. All procedures were performed for both sessions separately (Figs. 3D–F and 4A–C). To assess the reliability of scaling between sessions we overlaid the response amplitudes for both sessions on each other (Fig. 4G–I) and calculated the explained variance between sessions.

Additionally, we calculated the average z-scored response across cortical depth across all subjects and luminance contrasts. This average was converted from z-values to absolute % BOLD using the mean signal amplitude across subjects and contrasts and its standard deviation. The resulting curve was then scaled to a maximum of 1. This contrast-independent laminar response curve (*H*_*cir*_(*d*)) was then scaled to the original group-level responses to each of the presented luminance contrasts. We subsequently calculated the explained variance between the scaled contrast-independent laminar response curve and the original responses. This was repeated for all regions of interest and sessions (Figs. 3G–I and 4D–F).

## Supplementary information

Supplementary information.

## Data Availability

All data and code are available from the authors upon request.
