# Supplementary material for: Linear systems analysis for laminar fMRI: Evaluating BOLD amplitude scaling for luminance contrast manipulations
Source: Sci Rep. 2020 Mar 25;10:5462. doi: 10.1038/s41598-020-62165-x (PMC7096513; doi:10.1038/s41598-020-62165-x)
Supplement: Supplementary file 1 — Supplementary information. [file 41598_2020_62165_MOESM1_ESM.docx]

Linear systems analysis for laminar fMRI: Evaluating BOLD amplitude scaling for luminance contrast manipulations

# Supplementary Information

Jelle A. van Dijk^1,2,*^, Alessio Fracasso^2,3,4^, Natalia Petridou^4^, Serge O. Dumoulin^1,2,5^

^1^ Experimental Psychology, Utrecht University, Utrecht, NL; ^2^ Spinoza Centre for Neuroimaging, Amsterdam, NL; ^3^ Institute of Neuroscience and Psychology, University of Glasgow, Glasgow G12 8QB, UK; ^4^ Radiology Department, Imaging Division, Center for Image Sciences, University Medical Center Utrecht, NL; ^5^ Experimental and Applied Psychology, VU University, Amsterdam, NL

Corresponding author: Jelle A. van Dijk, [j.van.dijk@spinozacentre.nl](mailto:j.van.dijk@spinozacentre.nl)


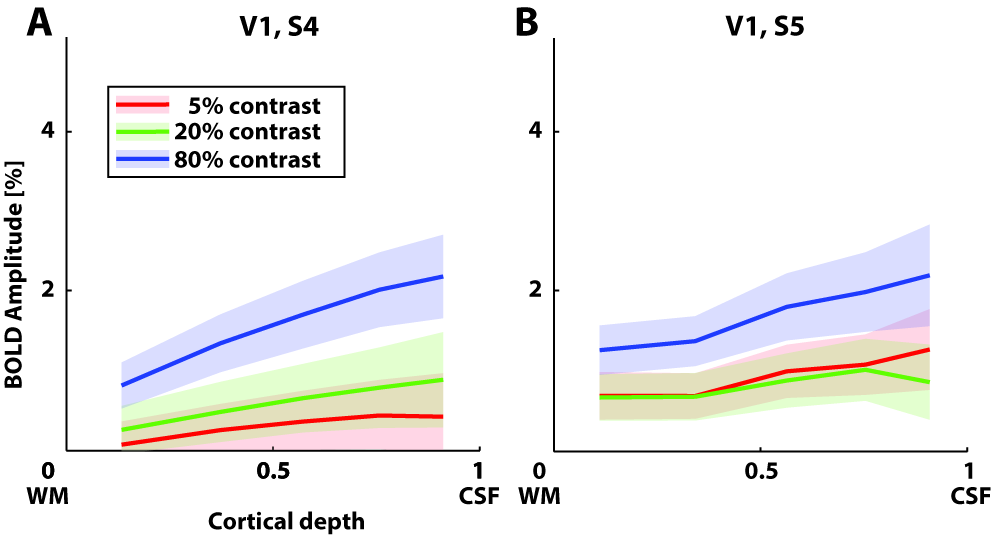


Figure S1: BOLD response amplitudes as a function of cortical depth and different luminance contrasts for the first session. BOLD response amplitudes across cortical depth for the two participants not shown in Figure 2A-C, for visual field map V1. Error regions represent 95% confidence intervals (+/-1.96 standard errors) of the mean across repeated stimulus presentations in a region of interest, for each depth bin and luminance contrast.


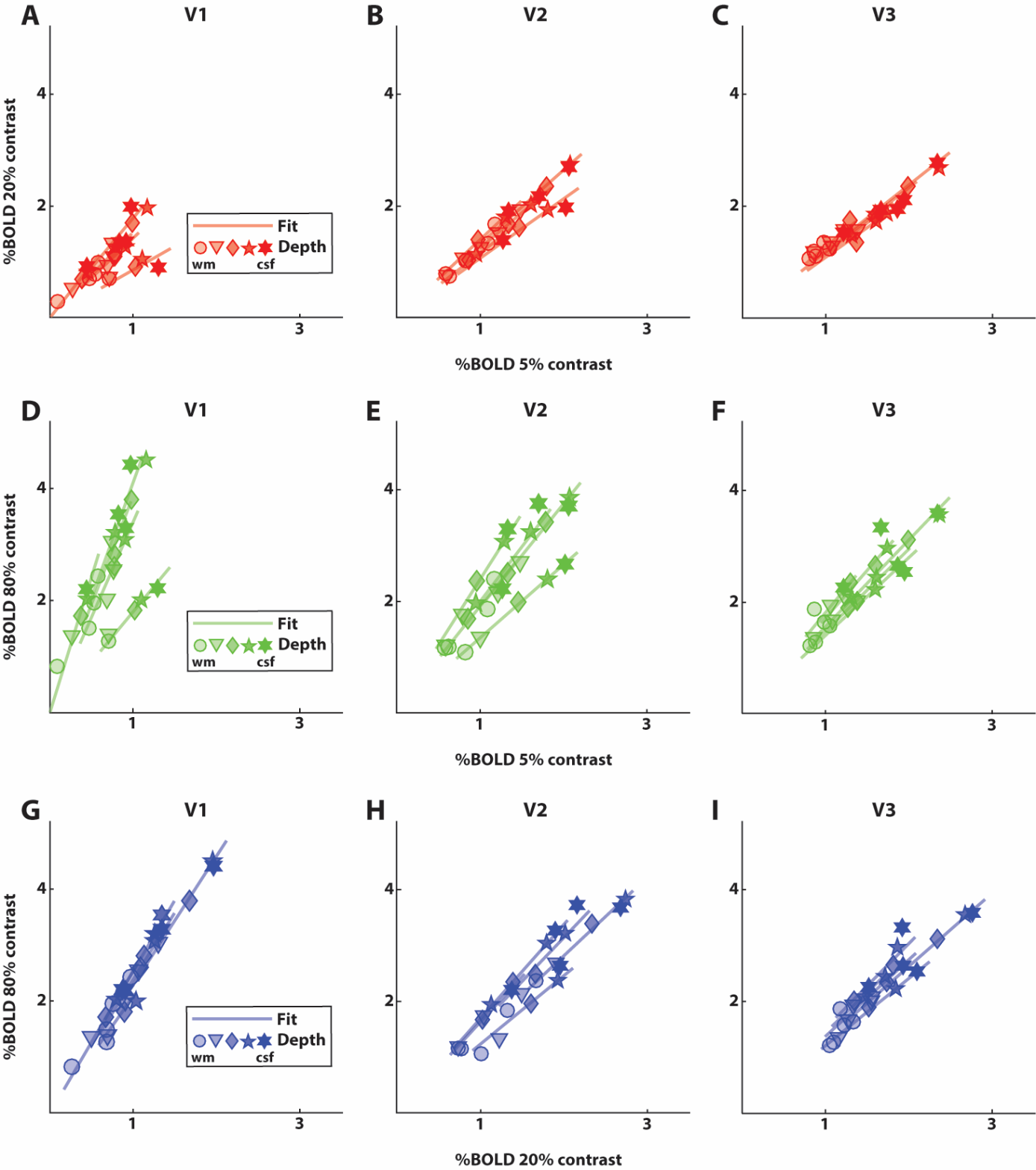


Figure S2: BOLD response amplitude at each depth bin for every combination of two luminance contrast presentations, for each participant, with best linear fit line. Different cortical depths are denoted as different marker shapes and transparency, as indicated in the appropriate legends. N.B. Combining each column of panels in one figure, results in Figures 3A-C. A-C) BOLD response amplitudes for 5% luminance contrast (x-axis) plotted against the BOLD response amplitude for 20% luminance contrast (y-axis) for V1-V3. D-F) BOLD response amplitudes for 5% luminance contrast (x-axis) plotted against the BOLD response amplitude for 80% luminance contrast (y-axis) for V1-V3. G-I) BOLD response amplitudes for 20% luminance contrast (x-axis) plotted against the BOLD response amplitude for 80% luminance contrast (y-axis) for V1-V3.


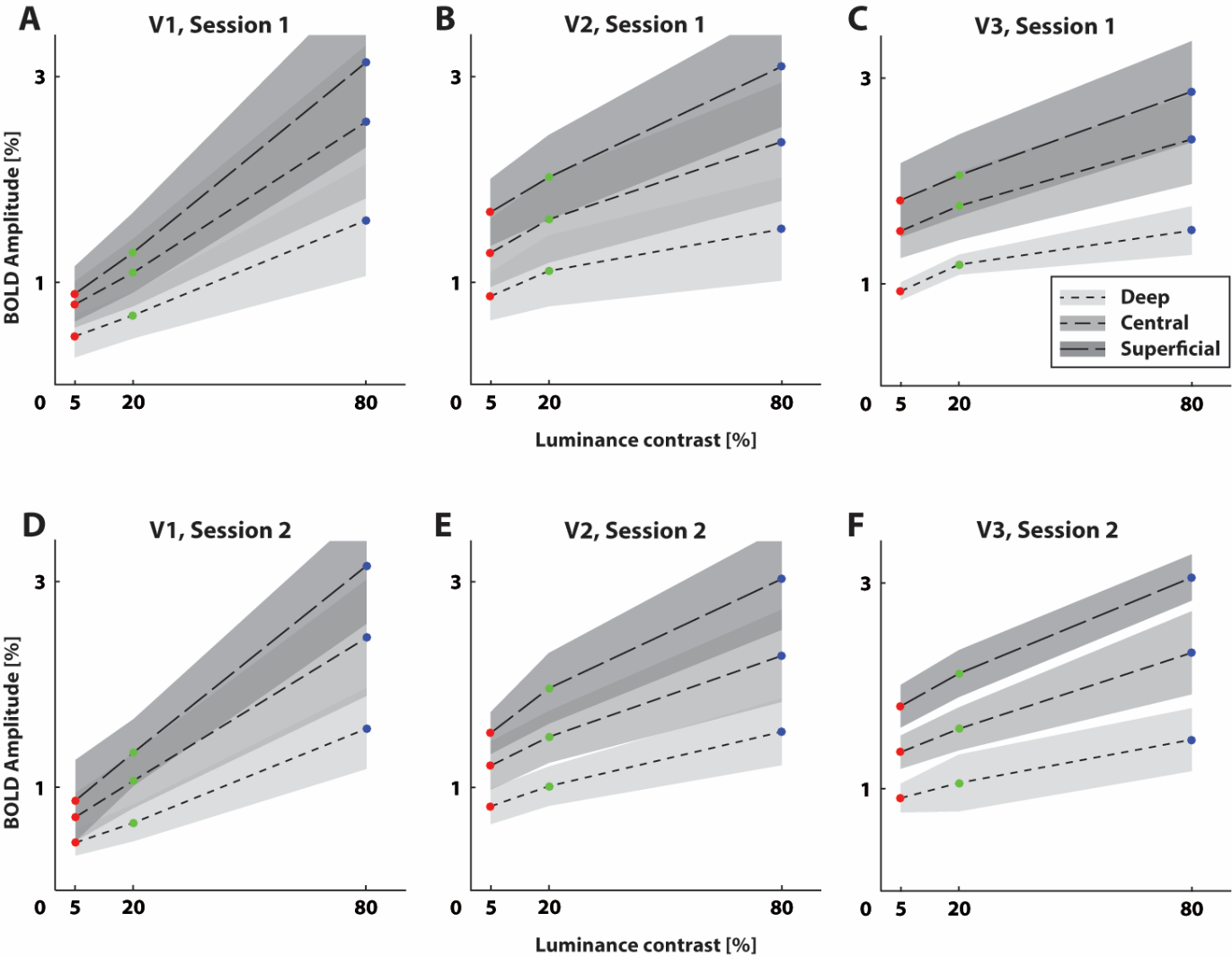


Figure S3: Average BOLD amplitude at three cortical depth bins (deep, central, and superficial) for 5%, 20%, and 80% luminance contrast. Dashed lines represent the between-subject mean responses. Shaded regions represent 95% between-subject confidence intervals. Marker colors correspond with luminance contrasts as presented in the main manuscript. Results for V2 and V3 show a nonlinear increase in BOLD amplitude with increasing luminance contrast, in line with the literature. Responses for V1 look to increase linearly with luminance contrast. However, as there are only few sampling points, this may not reflect the true response curve. A-C) All visual field maps, for session 1. D-F) All visual field maps, for session 2.
